# Spontaneous Bacterial Peritonitis and Henoch-Schönlein Purpura in a Patient with Liver Cirrhosis

**DOI:** 10.1155/2015/340894

**Published:** 2015-05-07

**Authors:** Neil Gupta, Joyce Kim, Basile Njei

**Affiliations:** Yale University School of Medicine, 333 Cedar Street, New Haven, CT 06510, USA

## Abstract

Henoch-Schönlein purpura (HSP) is a small vessel systemic vasculitis, predominantly affecting children, characterized by a tetrad of manifestations, specifically palpable purpura, arthralgia, abdominal pain, and renal disease. HSP in the adult population is rare, and no case has been described of HSP in liver cirrhosis with spontaneous bacterial peritonitis (SBP). We present the case of a 58-year-old male with liver cirrhosis, who was subsequently diagnosed with SBP and later HSP. In this patient, the diagnosis of HSP was demonstrated clinically by his palpable purpura, diarrhea, hematuria, and abdominal pain and confirmed pathologically by his renal and skin biopsies demonstrating leukocytoclastic vasculitis and IgA complexes. We believe that this is an example of altered IgA processing in cirrhosis leading to the development of IgA immune complexes and ultimately HSP. The patient additionally had SBP, which may have increased his risk for developing HSP given antigen processing by mucosa-associated lymphoid tissues leading to immune complex deposition, which may not have been effectively cleared in the context of his liver disease. The patient unfortunately died of gastrointestinal hemorrhage, which is unclear to be due to his underlying cirrhosis or a gastrointestinal manifestation of HSP itself.

## 1. Introduction

Henoch-Schönlein purpura (HSP) is a small vessel systemic vasculitis, predominantly affecting children, with ninety percent of cases occurring in the pediatric population. HSP is characterized by a tetrad of manifestations, specifically palpable purpura, arthralgia, abdominal pain, and renal disease, with gastrointestinal and renal involvement more prevalent in older adults [1, 2]. The characteristic finding is leukocytoclastic vasculitis accompanied by IgA immune complexes within affected organs. Gastrointestinal symptoms of HSP can range from mild symptoms of nausea, vomiting, or abdominal pain to gastrointestinal hemorrhage, bowel ischemia, and bowel perforation [3]. About half of cases of HSP are preceded by an upper respiratory infection or other underlying infections. HSP in the adult population is rare, and no case has been described of HSP in liver cirrhosis with spontaneous bacterial peritonitis (SBP). We present the case of a 58-year-old male with liver cirrhosis and SBP, confounded by the diagnosis of HSP.

## 2. Case Report

A 58-year-old male with past medical history of diabetes mellitus type 2, depression, HTN, chronic alcohol abuse, and BPH, presented to our hospital with worsening left-lower quadrant abdominal pain over three weeks and painless hematuria for five days. He had no known history of liver or kidney disease. Physical examination was significant for abdominal distension and left-lower quadrant abdominal tenderness. His initial labs demonstrated pancytopenia (white blood cell count 3.3, hemoglobin 7.7, hematocrit 23.4, and platelets 95), acute kidney injury (creatinine 1.6, unknown baseline), hypoalbuminemia (albumin 3.0), hyponatremia (sodium 129), and a nonanion gap metabolic acidosis (bicarbonate 15.5), Table 1(a). Urine studies showed large amount of blood and RBCs with some hyaline casts and proteinuria, and urine electrolytes showed FeNa of 0.8%, Table 1(d). CT abdomen showed a nodular margin of the liver with mild intrahepatic biliary dilation, suggesting chronic liver disease, along with splenomegaly. US abdomen confirmed heterogeneous and nodular contour of liver with splenomegaly and moderate amount of ascites, likely sequelae of cirrhosis, with dopplers negative for thrombosis. The nodular liver on CT, thrombocytopenia, hypoalbuminemia, splenomegaly, and moderate ascites all helped establish the new diagnosis of liver cirrhosis.

Despite further labs to exclude viral, autoimmune, and other causes, Table 1(b), the etiology of his liver cirrhosis remained unclear, attributable to NASH versus alcohol given his history of uncontrolled diabetes and chronic etoh abuse, Table 1(b). Diagnostic paracentesis was performed, showing glucose 182, LDH 79, albumin 0.9, and protein 1.7 with 6400 RBCs and 1850 nucleated cells (51% granulocytes, 17% lymphocytes, and 32% tissue cells), Table 1(c). Cytopathological examination revealed no neoplastic cells, and bacterial culture was negative. The corrected neutrophil count of 917 and serum-ascites albumin gradient of 2.1 were consistent with SBP and portal hypertension, Table 1(c).

After diagnosis of liver cirrhosis (Child-Pugh class B, MELD 6) and SBP, he was treated with IV ceftriaxone (2 g daily) and IV albumin (1.5 g/kg on first day and 1 g/kg on third day). He was placed on protonix given portal hypertension with unknown variceal status and was monitored for signs of hepatic encephalopathy, though he mentated well with no asterixis on exam. Repeat diagnostic paracentesis on day 3 demonstrated resolution of his SBP with 8150 RBCs and 305 nucleated cells (3% granulocytes, 51% lymphocytes, 45% tissue cells, and 1% eosinophils).

Despite the underlying diagnosis of liver cirrhosis and SBP, the basis of his renal dysfunction and hematuria continued to remain a mystery. Cystoscopy was negative for malignancy, and his urine sediment did not reveal any dysmorphic RBCs. Quantification of his proteinuria revealed a protein: creatinine ratio of 1.9. There was concern for hepatorenal syndrome (HRS) given his low FeNa and bland urine sediment. He was given albumin with no improvement in his renal function in addition to treating underlying SBP, while additional treatments such as midodrine and octreotide were not pursued given his high MAPs. Work-up for other causes of his renal failure continued.

**Figure 1:**
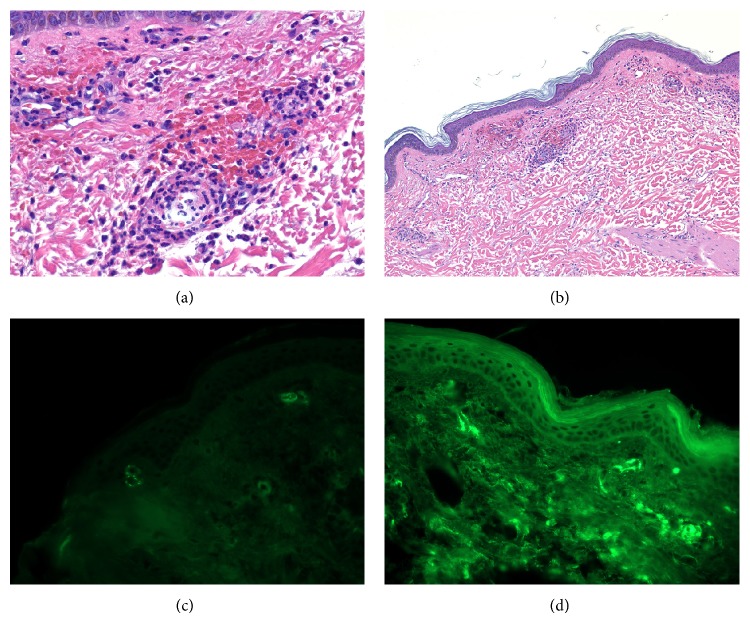
Skin biopsy, right arm. Top images are H&E slides of arm at high power (a) and low power (b), showing fibrin, neutrophils, and neutrophil fragments near vessels along with extravasated erythrocytes and a mixed cellular infiltrate. Bottom images are direct immunofluorescence (DIF) showing vascular wall staining with C3 (c) and IgA (d) supporting the diagnosis of HSP.

**Figure 2:**
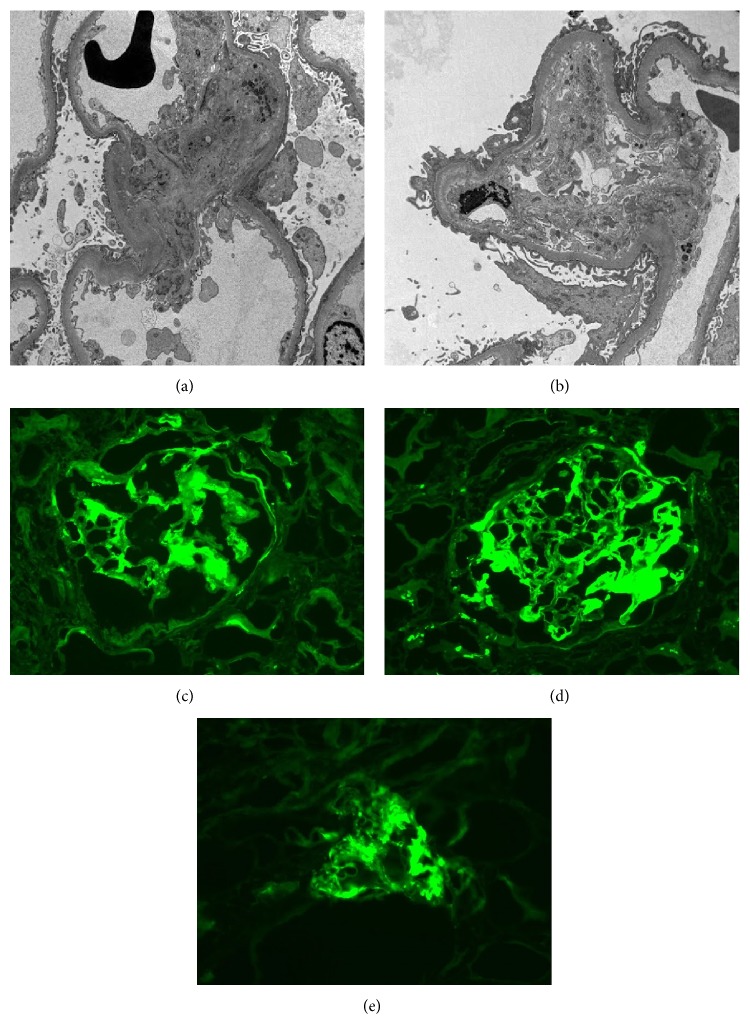
Renal biopsy. Top images (a and b) are electron microscopy slides demonstrating increase in mesangial matrix with segmental mesangial deposits. The glomerular architecture shows corrugation of basement membrane with focal effacement of foot processes. Bottom images are direct immunofluorescence (DIF) showing glomerular staining with C3 (c), fibrinogen (d), and IgA (e), supporting diagnosis of HSP.

Four days after admission, he had diarrhea, and thirteen days later, he developed new purpuric macules and papules, approximately 0.3–1.5 cm in size, most prominent on his elbows but also on his abdomen, buttocks, back, and inguinal folds. Skin biopsy showed fibrin, neutrophils, and neutrophil fragments near vessels with mixed cellular infiltrate and extravasated erythrocytes, Figures 1(a) and 1(b). Direct immunofluorescence (DIF) studies showed deposition of IgA and C3 in vessel walls, supporting the diagnosis of HSP, Figures 1(c) and 1(d). Due to worsening renal function with creatinine rising to 4.9, renal biopsy was performed revealing mesangial proliferative glomerulonephritis, Figures 2(a) and 2(b), with crescents, Figures 2(c) and 2(d), and IgA, Figure 2(e), also consistent with HSP. He was given one dose of solumedrol 500 mg IV and started on prednisone 60 mg, which was tapered to 50 mg daily, with protonix to decrease GI bleeding risk. The purpuric lesions subsequently resolved with corticosteroid treatment.

However, his renal failure continued to worsen with his creatinine rising to 5.1 and BUN to 157. Due to concern for worsening renal failure, uremia, and oliguria, he underwent emergent hemodialysis. His course quickly deteriorated as he became acutely hypotensive, with a sudden hemoglobin drop to 4.8. After two units of transfusion to hemoglobin of 8.8, he coded one day later with profuse hematemesis after an episode of bright red blood per rectum and died.

## 3. Discussion

In this patient, the diagnosis of HSP was demonstrated clinically by his palpable purpura, diarrhea, hematuria, and abdominal pain. The diagnosis was confirmed pathologically by his renal and skin biopsies demonstrating leukocytoclastic vasculitis and IgA complexes. While HSP is predominantly a pediatric disorder, its existence in adults is rare and its association with liver cirrhosis is even more scant [4–6]. There are only a few case reports describing the association between liver cirrhosis and HSP. For example, Aggarwal et al. reported one of the first cases of liver cirrhosis with acute renal failure and HSP [4]. Our case, however, is the first to show the significant extent of HSP in liver cirrhosis due to the combined renal and skin manifestations along with the additional complicating factor of SBP, which may have triggered his course of HSP.

Alcohol liver disease is characterized by IgA deposits in a continuous pattern along liver sinusoids, in addition to skin capillaries and mesangium of renal glomeruli. The liver contributes to the clearance of intravascular IgA, causing patients with chronic liver disease to show an increase in serum IgA concentration [7]. Van de Wiel et al. studied the presence and concentration of circulating IgA-containing immune complexes in patients with alcoholic liver disease and patients with other nonalcoholic liver diseases with comparable serum IgA levels [8]. He concluded that the presence of circulating IgA-containing immune complexes was directly related to the severity of liver damage and substantiated the pivotal role of the liver in clearing circulating IgA [8]. The role of liver cirrhosis in the development of HSP is intriguing since this patient's chronic liver disease may have precipitated the development of HSP with defective liver metabolism of IgA circulating immune complexes, leading to deposition in the skin and kidneys.

The diagnosis of SBP is particularly unique in this case and has not been previously reported in case reports of patients with combined HSP and liver cirrhosis. Observations in children with HSP have confirmed approximately 30–65% of IgA vasculitis cases occur after an upper respiratory tract infection [9]. Some propose that increased synthesis of IgA due to antigen processed by the mucosa-associated lymphoid tissue leads to development of the disease by immune complex deposition between antigens and IgA in the skin, gut, and kidneys [9]. In this particular case, it could be hypothesized that SBP may have triggered antigen processing by mucosa-associated lymphoid tissue in the gut and the subsequent development of IgA complexes, with impaired clearing due to the patient's underlying liver cirrhosis, leading to the significant extent of his HSP.

This patient unfortunately had a rapid decline due to his hospital stay. The extent of his HSP with combined renal and skin manifestations has previously not been described in the literature in association with liver cirrhosis. While he received corticosteroid treatment, this does not prevent nephritis or alter the course of HSP [10, 11]. His renal failure may have also been worsened by SBP and underlying cirrhosis, in addition to his IgA nephropathy. His ultimate hematemesis may have been due to variceal hemorrhage in the context of his liver cirrhosis, though gastrointestinal hemorrhage is known to be a rare manifestation of HSP.

## 4. Conclusions

This is a unique case of significant HSP with both renal and skin manifestations along with SBP in a patient with underlying liver cirrhosis. While HSP is rare in adults, we believe that this is an example of altered IgA processing in cirrhosis leading to the development of IgA immune complexes and ultimately HSP [7]. The patient additionally had SBP, which may have increased his risk for developing HSP given antigen processing by mucosa-associated lymphoid tissues leading to immune complex deposition, which may not have been effectively cleared in the context of his liver disease. The patient unfortunately died of gastrointestinal hemorrhage, which is unclear to be due to his underlying cirrhosis or a gastrointestinal manifestation of HSP itself.

## Figures and Tables

**(a):** 

Lab	Value	Reference
Wbc	3.3	(4–10)
Hg	7.7	(14–18)
Hct	23.4	(40–52)
Platelets	95	(150–350)
Na	129	(135–145)
K	3.7	(3.5–5)
Cl	98	(96–106)
HCO_3_	15.5	(22–30)
AG	16	(7–16)
BUN	26	(7–20)
Cr	1.6	(0.5–1.2)
Glucose	217	(70–100)
AST	49	(0–34)
ALT	31	(0–34)
ALP	184	(30–130)
GGT	253	(11–49)
DB	0.11	(>0.20)
TB	0.23	(<1.20)
Albumin	3.0	(3.5–5)
INR	0.92	(1-2)
Ammonia	72	(11–35)
AFP	1	(<6)

**(b):** 

Lab	Value	Reference
ANA	<1 : 40	(<1 : 40)
ANCA	negative	
Anti-DNASE B ab	<80	(0–200)
Antismooth IgG	13	(<20)
C3	86	(81–145)
C4	32	(16–39)
Ceruloplasmin	29	(18–51)
Ferritin	36	(30–400)
HgA1c	8.0	(4.0–6.0)
HIV	negative	
HbSAg	negative	
Hep C Ab	negative	
Hep BcIgM	negative	
Hep A IgM	negative	
LDH	181	(118–273)
Mitochondrial ab	4.7	(<20)
Ova/parasites urine	negative	
Schistosoma ab	0.95	(<0.20)
TSH	1.62	(0.27–4.20)
Total protein	5.9	(6.5–8.0)

**(c):** 

Lab	Value
Glucose	182 mg/dL
LDH	79 U/L
Protein	1.7 g/dL
Albumin	0.9 g/dL
RBC	6400 cells/uL
Nucleated cells	1850 cells/uL
Differential	51% granulocytes, 17% lymphocytes, and 32% tissue cells
Cytology	Negative for malignancy, mesothelial cells with histiocytes and neutrophils
Culture	4+ WBCs, no organisms

**(d):** 

Lab	Value
Protein	100 mg/dL
Blood	Large
Bilirubin	Negative
Leukocytes	Negative
Nitrites	Negative
Hyaline casts	3
WBC/HPF	17
RBC/HPF	1363
Urine Na	31 mmol/L
Urine K	17.9 mmol/L
Urine Cl	16 mmol/L
Urine Cr	50.7 mg/dL
Urine UN	666 mg/dL
FeNa	0.8%
